# Neuroendocrine Parathyroid Tumors: Quality of Life in Patients with Primary Hyperparathyroidism

**DOI:** 10.3390/biomedicines11072059

**Published:** 2023-07-21

**Authors:** Mara Carsote, Claudiu Nistor, Mihaela Stanciu, Florina Ligia Popa, Remus Calin Cipaian, Ovidiu Popa-Velea

**Affiliations:** 1 Department of Endocrinology, Faculty of Medicine, Carol Davila University of Medicine and Pharmacy & C.I. Parhon National Institute of Endocrinology, 050474 Bucharest, Romania; carsote_m@hotmail.com; 2 Department 4—Cardio-Thoracic Pathology, Thoracic Surgery II Discipline, Faculty of Medicine, Carol Davila University of Medicine and Pharmacy & Dr. Carol Davila Central Emergency University Military Hospital, 010825 Bucharest, Romania; ncd58@yahoo.com; 3 Department of Endocrinology, Faculty of Medicine, “Lucian Blaga” University of Sibiu, 550169 Sibiu, Romania; 4 Department of Physical Medicine and Rehabilitation, Faculty of Medicine, “Lucian Blaga” University of Sibiu, 550169 Sibiu, Romania; 5 Department of Internal Medicine, Academic Emergency Hospital of Sibiu, Faculty of Medicine, “Lucian Blaga” University of Sibiu, 550245 Sibiu, Romania; calin.cipaian@ulbsibiu.ro; 6 Department of Medical Psychology, Faculty of Medicine, Carol Davila University of Medicine and Pharmacy, 050474 Bucharest, Romania; opopavelea@hotmail.com

**Keywords:** quality of life, primary hyperparathyroidism, parathyroidectomy, hypercalcemia, score, SF-36, questionnaire, neuroendocrine, PTH, surgery

## Abstract

Tumors of the parathyroid glands, when associated with PTH (parathyroid hormone) excess, display a large area of complications; in addition to the classical clinical picture of primary hyperparathyroidism (PHP), a complex panel of other symptoms/signs can be identified, including memory and cognitive impairment, chronic asthenia/fatigue, reduced muscle functionality, depressive mood, non-specific bone pain, and loss of sleep quality. The perception of quality of life (QoL) can be supplementarily enhanced by their progressive onset, which makes many patients not be fully aware of them. Their improvement was reported very early after parathyroidectomy (PTx), yet the level of statistical evidence does not qualify these non-classical elements as standalone indications for PTx. Our objective is introducing an up-to-date on QoL scores with regards to the patients diagnosed with PHP, particularly taking into consideration PHP management from baseline to post-operatory outcome, including in cases with multiple endocrine neoplasia. This is a narrative review of literature. We revised full-length papers published in English through PubMed research conducted between January 2018 and May 2023 by using the key words “quality of life” and “primary hyperparathyroidism”. We particularly looked at data on self-reported QoL (through questionnaires). We excluded from the search the studies focused on non-PTH related hypercalcemia, secondary, and/or renal/tertiary hyperparathyroidism, and vitamin D supplementation. Overall, we identified 76 papers and selected for the final analysis 16 original studies on QoL and PHP (a total of 1327 subjects diagnosed with syndromic and non-syndromic PHP). The studies with the largest number of individuals were of 92, 104, 110, 134, 159, as well as 191. A few cohorts (*n* = 5) were of small size (between 20 and 40 patients in each of them). Concerning the study design, except for 2 papers, all the mentioned studies provided longitudinal information, particularly the timeframe from baseline (before PTx) and after surgery. The post-operatory follow-up was of 3–6 months, but mostly between 1 and 3 years (maximum a decade of surveillance). The age of the patients varies between medians of 56, 62, 64, and 68 years. Most frequent questionnaires were SF-36, PHPQoL, and PAS. Despite not being unanimously similar, an overall reduced score of QoL in patients with PHP versus controls was registered, as well as general improvement following PTx. Variations of QoL results might have a multifactorial background from different comorbidities, studied populations, technical aspects of collecting the data, etc. QoL scores in PHP represents a complex heterogeneous picture, from their correlation with clinical features and lab assays (e.g., the level of serum calcium), the associated comorbidities (such as multiple endocrine neoplasia syndromes), up to the assessment of the QoL improvement after parathyroidectomy (PTx). While current studies do not unanimously agree on each QoL domain, the assessment of QoL might represent a supplementary argument to consider when deciding for PTx, especially in asymptomatic cases and in patients who do not fit into well-known categories of surgery candidates, according to current guidelines, thus assessing QoL in PHP is part of a current research gap. QoL evaluation in PHP remains an open issue, towards which awareness should be cultivated by both endocrinologists and surgeons. The introduction of a routine evaluation of the QoL scores in patients, as well as the selection of the most appropriate questionnaire(s), represents an open chapter thus awareness in mandatory.

## 1. Introduction

Tumors of the parathyroid glands, when associated with PTH (parathyroid hormone) excess, display a large area of complications from cardiovascular issues to bone damage, from digestive complains to dramatic picture of acute hypercalcemia. In primary hyperparathyroidism (PHP), parathyroidectomy (PTx) represents the only curative option, being recommended in cases with specific entities such as osteoporosis and/or osteoporotic fractures, kidney stones, renal failure, or in patients younger than 50 years [1,2,3]. These well-established elements are both objective and easily identifiable, when selecting candidates for PTx. Due to the current excellence in calcium screening protocols, currently, in developed countries, most of the PHP cases are asymptomatic, requiring long-term surveillance. Among the “non-surgical” individuals, at least one out of four will become symptomatic within the next 15 years since first diagnosis of PHP, with the patient being eventually referred for PTx [4,5,6].

In addition to the classical clinical picture of PHP, a large panel of other symptoms/signs can be identified, including memory and cognitive impairment, chronic asthenia/fatigue, reduced muscle functionality, depressive mood, non-specific bone pain, and loss of sleep quality [6,7,8,9]. As a whole, they fall into one of the categories: neuropsychiatric/psychological, cardiovascular, articular, and muscular symptoms, and may be mistaken by patients as pertaining to the physiological aging process or to the daily stressful activities. This perception can be supplementarily enhanced by their progressive onset, which makes many patients not fully aware of them [6,8,10]. Their improvement was reported very early after PTx in some patients, yet the level of statistical evidence does not qualify these non-classical elements, including impairment of quality of life (QoL), as recommendations for PTx, this being the reason for not including them in current guidelines [6,8,11]. 

Whether abnormal QoL scores represent an indication of PTx is still an open issue; so far, it appears that we need more evidence on PHP-related QoL, in addition to the traditional panel of assessments such as bone profile for osteoporosis confirmation and screening of fragility fractures, imaging evaluation for kidney stones, and renal function assays in order to provide a better understanding of the disease and thus an optimum management [2,8]. 

### Aim

Our objective is introducing an up-to-date on QoL scores with regards to the patients diagnosed with PHP, particularly taking into consideration PHP management from baseline to post-operatory outcome, including multiple endocrine neoplasia.

## 2. Methods

This is a narrative review of the literature. We revised full-length papers published in English through PubMed research conducted between January 2018 and May 2023, by using the key words “quality of life” and “primary hyperparathyroidism”. We particularly looked at data on self-reported QoL (through questionnaires). To improve both relevance and accuracy of the analysis, we excluded from the search the studies focused on non-PTH-related hypercalcemia, secondary and/or renal/tertiary hyperparathyroidism, and vitamin D supplementation. 

Inclusion criteria of selected articles were *in extenso* original studies (at least 20 patients per study) that were published in English, with PubMed access, timeline between January 2018 and May 2023; articles that were identified according to mentioned research key words strategy in title and/or abstract; cross-sectional or longitudinal studies in humans with any level of statistical evidence, either of retrospective or prospective design; respectively, syndromic or sporadic PHP. Exclusion criteria were: case reports; case series; reviews; editorials; letters to editors; studies on patients with different conditions other than PHP such as hypercalcemia of other causes; chronic renal failure or osteoporosis; other types of diseases with an increased level of PTH like secondary, renal, tertiary types; studies that did not include patients with data concerning self-reported questionnaires that involved the assessment of QoL. 

**Figure 1:**
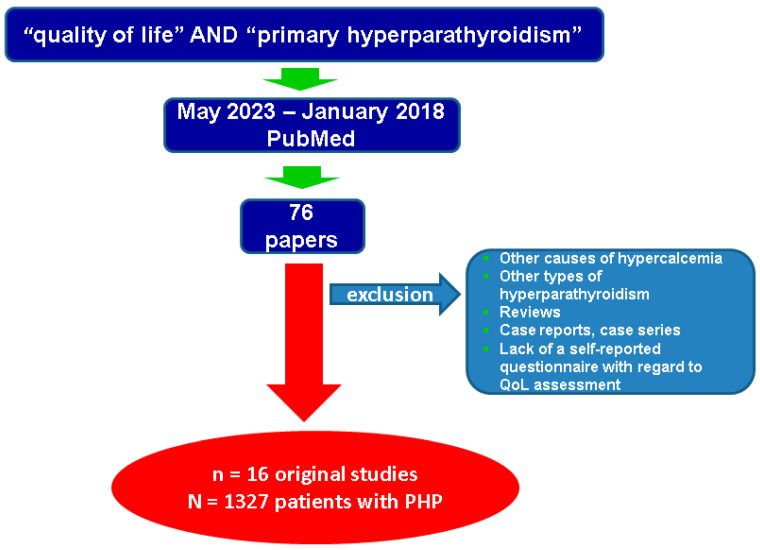
Flow chart diagram according to mentioned methods. Abbreviations: QoL = quality of life; *n* = number of articles; N = number of patients; PHP = primary hyperparathyroidism.

Each study was objectively selected; thus, overall, we identified 76 papers according to mentioned methods and selected for the final analysis 16 original studies on QoL and PHP (a total of 1327 subjects diagnosed with syndromic and non-syndromic PHP and evaluated through a self-reported questionnaire of QoL) (Figure 1).

## 3. Results: QoL in Patients Diagnosed with Parathyroid Tumors

### 3.1. QoL Scores among the Panel of Investigations in Primary Hyperparathyroidism

The articles that met the inclusion/exclusion criteria used a series of QoL questionnaires, such as the Short Form 36 Health Survey Questionnaire (SF-36), Pasieka’s Parathyroid Assessment of Symptoms Questionnaire (PAS-Q), the Comprehensive Psychopathological Rating Scale (CRPS), the Primary Hyperparathyroidism Quality of Life (PHPQoL) questionnaire, and the EQ-5D-3L health status questionnaire with two components: five domains on physical and mental health (MH) and visual analogue scale (VAS) [12,13,14]. 

Among the majority of studies, the most used instrument was the SF-36, which has been widely acknowledged as being one of the most user-friendly and reliable tools for assessing physical health (PH) and MH. This test comprises 36 questions, concerning two main components: PCS (Physical Component Summary), which reflects, in its turn, the mean score of four domains—PF (physical functioning), RP (role-functioning physical), BP (bodily pain), and GH (general health)—and MCS (Mental Component Summary), which is compiled from the mean score of other four domains—VT (vitality), SF (social role functioning), RE (role-functioning emotional), and MH. Each domain is quantified by a 0–100 scale (a higher score means a better QoL). The procedure for collecting data is based on self-reported answers [15,16]. 

### 3.2. The Influence of Parathyroidectomy on QoL

As expected, after the stable correction of calcium and PTH levels provided by the PTx, both QoL components (PCS and MCS) are generally reported to increase. Still, the comparative PTx impact on different QoL subscales seemed to be not homogenous [6]. In this respect, Somuncu et al. [14] published a study in 2021 on 41 candidates to PTx who were confirmed with PHP (median age of 64 years, ranges between 32 and 83). SF-36 and PHP-QoL showed a significant improvement pre-PTx versus 12 months post-PTx (*p* < 0.05), ranging from 50% for physical domains to 76% for MH [14]. 

Similar data were revealed by Christensen et al. [17] in a prospective study from 2022 on 40 patients with PHP (median age of 62 years, ranges between 28 and 82; 65% were females). Out of the eight domains of SF-36, six categories showed a statistically significant increase 6 months post-PTx, three of them concerning PCS: VT (*p* = 0.0001), PF (*p* = 0.038), RP (*p* = 0.043), and another three concerning MCS: GH (*p* = 0.004), SF (*p* = 0.004), MH (*p* = 0.0001) [17]. Interestingly, some of the patients were enrolled during the COVID-19 pandemic, which did not seem to affect the study results [17]. However, the clear overall effects of lockdown regulations on QoL scores and other complications of PHP amid the pandemic are yet to be determined [18]. 

QoL score dynamics after PTx were found to be dependent on the patients’ age. Papavramidis et al. [19] identified through one prospective cohort that both the younger group diagnosed with PHP (≤65 years; N = 95, mean age of 50.4 ± 9.8 years) and the older group with PHP (of 65 years and older, N = 38, average age of 72.1 ± 4.9 years) had a statistically significant PAS-Q score improvement from pre-PTx to 6 months post-PTx, but the underlying best parameters were different among the two groups (for example, “itchy skin”, “mood swings”, “irritability”, and “feeling thirsty” [19] were found more often in the younger group, while “bone pain”, “tiredness”, “weakness”, “joint pain”, “getting off chair”, and “headaches” [19] were identified more frequently in the second group) [19]. Also, only the older group had a significant post-operatory decrease of the frailty index, suggesting that age is a parameter to consider when taking into consideration the extent of PTx impact on QoL [19].

Post-PTx QoL changes were described also in asymptomatic PHP (asymptomatic PHP does not necessarily mean that QoL parameters are not affected, but the data we have so far are heterogeneous). Thus, one study on 78 patients with PHP (median age of 62 years, 72% were females) was able to identify an early post-PTx global improvement of the VAS score (EQ-5D-3L health status questionnaire) in both symptomatic PHP (N = 56, *p* < 0.001) and asymptomatic PHP (N = 28, *p* = 0.014). Additionally, Vadhwana et al. [12] showed that the symptomatic group attained a decrease in their post-PTx anxiety and depression levels (*p* < 0.01) [12].

### 3.3. Conservative Approach in Primary Hyperparathyroidism and QoL

PTx contribution to QoL improvement versus a conservative approach typically was taken into consideration for those patients who were not immediate candidates for surgery, according to standard guidelines (by not having the criteria or by displaying severe co-morbidities that required a delay of the surgery) [6]. The impact of conservative management in PHP was assessed based on longitudinal data or with regard to the impact of PTx-associated analysis or by comparing the subjects with normal (non-PHP) population [13,15,20,21,22,23,24,25].

The largest cohort on this particular matter was described in the SIPH (Scandinavian Investigation on Primary Hyperparathyroidism) [13] study. This was an international, multi-center, a 10-year longitudinal, randomized, prospective study on mild PHP, comparing patients referred to PTx (N = 95) to those under conservative surveillance (N = 96). The PTx group significantly improved VT score after a decade (*p* = 0.017) versus the non-PTx group, which did not change any domain of SF-36. CRPS showed an improvement in both groups versus their baseline values, while finding no significant difference between them. The study concluded that QoL assessment should be integrated to usual bone and renal assays in long-term decisions in individuals with mild PHP [13].

Another study providing a long-term analysis was published in 2019 by Tzikos et al. [25] and confirmed the QoL benefits of PTx over non-PTx in asymptomatic PHP. The participants who were either treated with PTx (N = 18) or conservative (N = 20) had a PAS-Q total score before surgery decrease 3 months post-PTx (*p* = 0.003), meaning a QoL improvement, respectively, 3-year post-PTx (*p* = 0.001) when compared to the second group where PAS-Q score increased versus baseline (*p* = 0.019) and it became higher than in the PTx group after 3 years (*p* = 0.021) [25].

### 3.4. Mild/Normocalcemic/Asymptomatic Forms of Primary Hyperparathyroidism

One of the greatest challenges in dealing with the new face of PHP (mostly, asymptomatic due to early detection with a low risk of bone and renal complications) is recognizing that QoL is potentially affected despite not registering the classical features of complications [12,20,21].

A study on mild PHP (mean age of 68 years) included normocalcemic patients with PHP (N = 32) and hypercalcemic subjects diagnosed with PHP (N = 82). Both groups reported after 12 months from the PTx a QoL improvement (SF-36) in the physical domains (*p* = 0.04, and *p* = 0.016, respectively). Still, regarding the MH component, only the second group confirmed the benefits of PTx in terms of QoL (*p* = 0.043) [20].

Mild PHP (defined as values of total calcium no higher than 1 mg/dL above the upper limit, without classical symptoms of PHP) was the topic of a systematic review published in 2020. This paper identified four randomized controlled trials and six observational studies that confirmed post-PTx improvement of QoL scores (7/10 used SF-36). Still, the clinical relevance of each QoL domain has been less understood in individuals with PHP underlying different degrees of severity, especially in poorly symptomatic or asymptomatic cases [21].

As noted before, the frame of “mild PHP” is defined by a fine line, this term sometimes overlapping “asymptomatic” or “normocalcemic” PHP [22,23]. The assessment of QoL might play a more important role in cases with PHP who are under surveillance rather than in individuals with well-established indications for PTx. All mentioned studies showed that the subgroup of subjects who are referred to surgery associate a post-operatory improvement of QoL scores on different domains (there is not a general). This in turn may raise the question of reconsidering the PTx strategy, probably by refining the stratification of risks [21,22,23].

### 3.5. Primary Hyperparathyroidism-Related Serum Calcium and QoL Influence

As previously mentioned, PTH-dependent hypercalcemia displays a certain influence on QoL, but not all studies agree on this matter [17,24,25]. For example, Christensen et al. [17] did not identify a significant relationship between QoL and calcium levels dynamics, as oppsed to other researchers. In the already-mentioned study of Tzikos et al. [25], the non-PTx group with asymptomatic PHP had baseline total calcemic directly correlated to PAS-Q scores (r = 0.524, *p* = 0.018). However, in opposition to the results of SF-36, higher PAS-Q scores were associated with a poorer QoL [25].

PAS-Q and PHPQoL were applied pre-PTx and 12 months post-PTx in a prospective cohort study published by Ejlsmark-Svensson et al. [24]. A total of 104 individuals with PHP were included (median age of 64 years; 73% were females). Ionized calcium and PTH negatively correlated with PHPQoL (*p* < 0.04), which was different between subjects with mild versus moderate-severe hypercalcemia (*p* = 0.01). PAS-Q and PHPQoL were similar between patients with and without PHP-related osteoporosis, kidney stones, and renal dysfunction; however, they were different pre-PTx versus post-PTX (*p* < 0.02), confirming post-operative QoL improvement. This study showed that hypercalcemia influences QoL more, compared to specific PHP-related organ complications. Of note, the authors used ionized serum calcium assays, while most of the data applied the total level of calcium [24]. 

Mohan et al. [26] published in 2021 a study on 50 patients with symptomatic PHP (mean age of 40.8 ± 13.9 years, 60% were females), who were grouped in four categories depending on total calcium levels at PHP diagnostic. The authors noted an overall improvement of QoL scores (SF-36), from pre-PTx to 3 months post-PTx (*p* < 0.001), especially for BP, RP, VT, and MH. MH scores improvement was dependent on pre-PTx calcium levels, while PH scores were not. Initially, QoL was more affected in subjects with severe hypercalcemia, but the other groups were affected to some extent, too. The relationship between blood calcium and QoL scores, including post-PTx improvement, suggested that QoL evaluation might represent a factor in surgical management, including the early decision to rapidly correct hypercalcemia [26].

High levels of calcium also involve muscle weakness, as a special contributor to QoL deterioration. One small study on menopausal women (N = 13 with normocalcemic PHP and N = 7 with hypercalcemic PHP) confirmed reduced physical performance and strength in both groups. In this study by Voss et al. [27], GH (SF-36) was impaired in normocalcemic patients, and MH (SF-36) was affected in hyperglycemic subjects, suggesting a more complex influence of the QoL, which cannot be fully captured by simple calcium assays [27]. 

The calcium–QoL relationship seems to be multifactorial, which explains the heterogeneity of the abovementioned results. Another confounding factor involves serum calcium assays variability, which depends on the degree of hydration/fluid replacements, renal dysfunction, or exposure to various medications. Concerning the latter, intravenous bisphosphonates, denosumab for acute hypercalcemia, as well as other calcium-lowering drugs such as cinacalcet or long-term medication against osteoporosis and fracture risk prevention in PHP may all display a strong influence on serum calcium, especially within the first days following the injection [3,28]. 

### 3.6. QoL in Syndromic (Heritable) Primary Hyperparathyroidism 

Syndromic PHP includes, among others, multiple endocrine neoplasia type 1 and 2A, which brings to the surface additional issues related to other non-parathyroid neuroendocrine tumors such as medullary thyroid carcinoma, pheochromocytoma (for type 2A), gastro-entero-pancreatic, and pituitary neuroendocrine neoplasia (for type 1) [29,30,31,32]. As expected, QoL represents the output from several conditions, thus specific influence of high PTH and calcium and associated co-morbidities should be integrated to the larger frame of genetically related tumors/malignancies [33,34,35]. However, the level of evidence concerning QoL in this specific matter remains low. 

We identified one prospective study from 2022, addressing the influence of PTx on QoL scores (SF-36) in subjects with multiple endocrine neoplasia type 1-associated PHP who were PTx candidates. Brescia et al. [36] included 30 patients (median age of 38 years, ranges between 28 and 44) who had total PTx with autograft (N = 20) and subtotal PTx (N = 10). Asymptomatic PHP (N = 19) versus symptomatic PHP (N = 11) correlated with a higher PCS (*p* = 0.0051), respectively, a higher MCS (*p* = 0.04), and a lower age (30 versus 38 y, *p* = 0.04). A total of 63.3% of subjects had pre-operatory bone pain; pain score did not correlate with patients’ age or with biochemical/hormonal assays, except for alkaline phosphatase (r= −0.44, *p* = 0.0162), but with PCS (r= −0.6, *p* = 0.0004) and MCS (r= −0.57, *p* = 0.0009). PCS and MCS were similar before and after PTx, regardless the number of PHP-associated comorbidities; the only domain with marginal significance was SF (69 versus 82, *p* = 0.04). Overall, median QoL scores were stationary from 6 months pre-PTx to 12 months post-PTx, but underlying a different individual variability among SF-36 parameters. Pre-operatory moderate/severe pain was more frequent in patients who developed post-surgery hypoparathyroidism. The frequency of low post-operatory PTH status was similar in individuals with pre-surgery asymptomatic and symptomatic PHP (27% and 21%, respectively); the subjects who developed hypoparathyroidism were older (*p* = 0.02) when compared to those who did not experience low PTH following PTx. QoL was not affected by the presence of post-operatory hypoparathyroidism or by the type of PTx that have been performed for PHP. Marginal significance was found between PCS and parathyroid remnant volume at 6 and 12 months post-PTx, respectively. Studied patients had different combinations of tumors among multiple endocrine neoplasia type 1-associated panels. When comparing the individuals with 1–2 comorbidities (N = 21) to subjects with 3–4 syndrome-associated comorbidities (N = 9), the first group had a higher PCS pre- and post-PTX, and better MCS at 12 months post-PTx. Within each group, PCS and MCS were similar pre- and post-PTx [36]. These results only partially confirmed the QoL scores improvement after PTx but showed the influence of non-PHP disorders in individuals confirmed with multiple endocrine neoplasia syndromes.

## 4. Discussions

### 4.1. Limits of Addressing QoL in Subjects with Primary Hyperparathyroidism 

Studies on PHP-related QoL are limited and typically comprise small sample size (from 20 to almost 200 subjects per paper) [12,13,14,17,19,20,24,25,26,27,36]. In terms of both methodology and results, these data represent a heterogeneous picture so far, as they include multiple elements with potential impact on QoL (from clinical presentation, e.g., bone pain, fractures, kidney stones, to the content of the surgical procedure itself, post-operative events, and characteristics of the non-PHP reference population—e.g., age, other comorbidities, etc.) [37]. 

An important limit of applying QoL scores is represented by the scales validation in each native language, an element that is paramount for their routine use in daily practice [38]. In this respect, National Societies of Endocrinology and Surgery should be further encouraged to perform validation studies of QoL questionnaires, potentially bringing valuable multicenter data on PHP-related QoL. The most relevant QoL scale in PHP patients is still a matter of debate, especially since the application of different questionnaires (such as SF-36 and CRPS) on the same population might display different results, as pointed by the outcomes of the SIPH study [13]. Aiming for the construction of a unitary instrument for assessing QOL in PHP seems, however, to be the next logical step, as various studies show a variable degree of consistency between the results provided by different instruments (SF-36 and PHPQoL [14] or PAS-Q and PHPQoL) [24]. Some studies did not apply classical QoL questionnaires, but monitored nonspecific symptoms, such as chronic fatigue or asthenia. These symptoms, despite being rather challenging to be quantified, represent an important non-specific clinical attribute of PHP and of PHP-related QoL [7,10]. For example, one study by Boone et al. [39] from 2017 showed on 20,091 consecutive patients with PHP that approximately 72% of them had chronic fatigue regardless the serum calcium levels [39].

The use of self-reported QoL questionnaires might vary with the severity and the duration of PHP. The timing of QoL improvement after PTx is not clear, the majority of the studies focusing on the first 3-6-12 months, while the most extended cohorts included a 3-year up to a 10-year period (such as SIPH study) [13,25]. The exact influence of either high calcium levels, increased PTH values, or both of these assays as well as the already established traditional comorbidity panel (like osteoporosis, kidney stones, renal failure, cardiac complications, etc.) on QoL scores is yet to be determined, representing a part of parathyroid disorders-associated challenges [40].

When comparing the conservative approach versus PTx, there is a subgroup of patients who are offered for a limited period of time therapy with calcimimetics as part of the medical management in PHP, an alternative to surgery in subjects refusing PTX or displaying contraindications to surgery [3,41,42,43,44,45]. One study correlated their use with QoL profile. Koman et al. [46] showed that control of calcium levels by using calcimimetic treatment (cinacalcet 30–60 mg per day) for 4 weeks before PTx improved QoL scores and predicted a better post-PTx outcome [46]. This was an observational study (N = 110, median age of 62 years) that applied different scales before cinacalcet use, one month after this therapy was offered to the patients and following PTX at 6 weeks as well as at 6 months. The subgroup that improved QoL under this medication before surgery correlated with a better post-operatory outcome that an interesting prediction tool might be provided by the cinacalcet-associated QoL [46].

In subjects with PHP and severe vitamin D deficiency, careful VD supplementation is advised, in order to improve the post-operatory outcome, especially muscle-skeletal health, and to correct the component of secondary (hypovitaminosis D-related) hyperparathyroidism [3,47,48,49,50]. Additionally, VD replacement might impact muscle strength and different other aspects of QoL scores, both on physical and general wellbeing perspectives [51,52].

### 4.2. Co-Morbidities in Primary Hyperparathyroidism: QoL Reflection 

As mentioned, in syndromic PHP, QoL displays a multifactorial influence. This includes the presence and the management of other (non-parathyroid) multiple endocrine neoplasia and associated complications, with a high medical and surgical burden [53,54]. One cross-sectional study from a national Dutch MEN1 cohort (N = 227) showed a reduction of QoL scores (SF-36) in individuals diagnosed with this syndrome versus the general Dutch population, the main predictors being the current employment status, respective the diagnostic of a pituitary tumour [55]. Another study showed that adults with multiple endocrine neoplasia type 2A (N = 45) had a reduced QoL score in all seven domains (based on Patient-Reported Outcomes Measurement Information System 29-item questionnaire) versus US normative data, displaying some similarities or even worse profile than other chronic conditions [56]. 

PHP-related QoL score seems to be influenced by the number of the syndromic tumors and associated complications in addition to advanced age at the moment of PTx and the presence of symptomatic (pre-operatory) PHP, especially bone pain [36]. Multiple endocrine neoplasia type 1-related PHP (which is mostly referred to PTx) raises the question of exact timing of PTx especially in asymptomatic, normocalcemic cases and young subjects, and QoL scores might represent an additional useful tool in order to decide the moment of intervention [57]. 

Due to genetic background causing synchronous or asynchronous parathyroid hyperplasia (which has been recently renamed by the World Health Organization “PHP-related multi-glandular parathyroid disease”), the parathyroid glands surgery typically involves a more complex procedure, for instance, with autograph [58,59,60]. Some authors reported a higher risk of post-operatory hypoparathyroidism in hereditary forms of PHP undergoing surgery, probably caused by an intense neck exploration [61,62]. Post-operative persistent low values of serum calcium and PTH might be an additional contributor to QoL scores deterioration [36,62,63,64]. Hillary et al. [65] suggested that overall QoL score might not be affected in post-PTx hypoparathyroidism, but rather isolated components such as chronic fatigue.

Interestingly, the first study to address HRQOL in patients confirmed with multiple endocrine neoplasia type 1 was published in 2018 by Peipert et al. [66]. The authors showed that PROMIS score (Patient-Reported Outcomes Measurement Information System via 29-item profile) was lower in these patients with regard to many components such as anxiety, depression, pain, fatigue, etc. when compared to normative data, but these patients also displayed decreased scores versus patients diagnosed with other cancers or neuroendocrine tumors (specifically for anxiety, depression, and fatigue) [66].

### 4.3. Integrating the Assessment of QoL among Pre-Operatory Panel of Evaluation in Primary Hyperparathyroidism

Routinely applying a QoL assessment based on self-reported questionnaires at first admission for PHP or before PTX is yet to be implemented in daily practice. Some clinical elements such as muscle weakness or joints/bones non-specific pain may be evident, while others such as depression or feeling “blue” might be underestimated by a usual endocrine check-up, although other hormone anomaly-associated conditions might be associated with a similar presentation (for example, severe hypothyroidism or Cushing’s syndrome) [67,68]. Concerning PHP, we mention the study from 2023 of Ionova et al. [69] that showed that 88% of the 92 individuals with PHP (median age of 56 years, mostly females) had such complaints. The authors also revealed that SF-36-based QoL was statistically significantly lower than the general (non-PHP) population (*p* < 0.001), while one out of ten subjects fit into the category of a very poor QoL score. A significant improvement of QoL was registered following PTx, both in terms of SF-36 (except for BP), and PHPQoL, when comparing to the pre-operatory status [70]. Generally, PTx represents the only curative option in PHP [71,72]; however, a subgroup of patients with pre-operatory depression should be specifically addressed and followed until complete remission, which might take a few weeks until a few months after surgery (with an adjustment of lifestyle intervention and specific medication, if needed). 

Another interesting parameter is represented by the sleep quality in patients with PHP. Febrero et al. [73] studied 65 subjects with PHP by applying Beck-II and Pittsburg questionnaires for sleep and depression evaluation in addition to SF-36 and PHPQoL. As seen with other parameters, sleep quality was statistically significantly affected in PHP versus controls (*p* < 0.05) [73]. Overall, most QoL components improved after surgery, but some authors suggested better results for physical components than mental ones, which did not reach the levels identified in the general population, as seen, for instance, in the study of Frey et al. [74] (N = 159 subjects with PHP, mean age of 62.6 years) [74]. Of note, QoL might be influenced by educational level, as well, in subjects confirmed with PHP, this being another factor to be taken into consideration as part of the general management in PHP [73].

### 4.4. Longitudinal Data on PHP: Should QoL Assessment Find Its Way?

Longitudinal studies in PHP are mostly focused on post-operatory outcome between 1 and 3 years (up to 10 years) [13,75]. When assessing the early post-operatory QoL, the impact of PTx itself should be addressed [37,76,77]. A massive progress has been registered during the modern era of parathyroid surgery regardless of the initial clinical picture and associated complications/co-morbidities as seen in multiple endocrine neoplasia [53,77,78]. 

Self-reported questionnaires to evaluate QoL are not mandatory in each patient with PHP according to the current protocols and guidelines [71,79,80]. However, a large amount of evidence is published so far that continues to raise the question of their practical utility. One of the largest analyses on published studies was reported in 2022, and it included 31 studies from 14 countries (a study between 1998 and 2021). Livschitz et al. [81] introduced in their systematic review 3298 subjects with PHP and more than 5000 individuals as controls (with non-PHP conditions). A total of 68% of these papers used SF-36, and 26% of them applied PAS to address QoL in PHP. The median of post-PTx follow-up was of 12 months (between 1 and 10 years). A total of 87% of these studies confirmed the improvement of QoL following parathyroid surgery [81].

PTx remains the single curable option in PHP thus providing an overall good outcome currently [82,83,84]. QoL, as similarly debated other elements (including age), might be a matter of new criteria for PTx candidates in asymptomatic PHP, too [63,82]. According to current guidelines, QoL impact does not stand alone as a PTx indication unless other well-established criteria of surgery are already identified. It seems that it is actually not the lack of evidence in QoL-PHP matter that represents the main issue, but rather the lack of standard routine application of such questionnaires mainly by endocrinologists and surgeons who are taking care of the individuals diagnosed with PHP. That is why a rather personalized approach of QoL topic might be useful [1,85,86].

**Figure 2:**
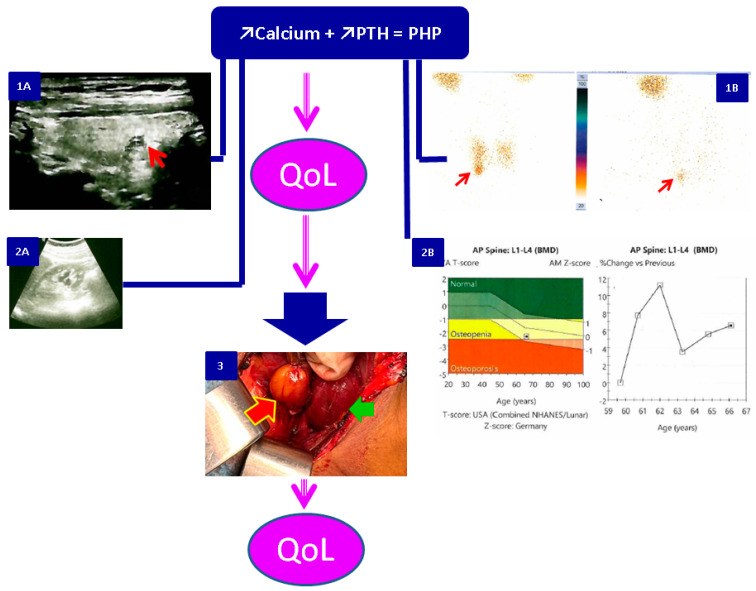
Potential use of QoL assessment based on self-reported questionnaires amid overall management of primary hyperparathyroidism: on admission, before, and after surgery. The diagnosis of PHP is based on high calcium and PTH levels; it is followed by imaging procedures to locate the tumour (1A = anterior cervical ultrasound; red arrow shows a right inferior parathyroid adenoma of 1.1 by 0.77 cm; 1B = Technetium Tc^99m^ parathyroid scintigraphy; red arrow shows the same adenoma at different moments of tracer capture); traditional complications of PHP should be checked, too, such as renal stones (2A = kidney ultrasound on the same 66-year-old female with PHP) and central Dual-Energy X-ray Absorptiometry (DXA) that shows osteoporosis on the same woman according to a lumbar L2–4 score of −2.5 SD. Intra-operatory aspect (3) introduces the parathyroid adenoma (red arrow; green arrows shows right thyroid lobe). Abbreviations: QoL = quality of life; PTH = parathyroid hormone; PHP = primary hyperparathyroidism.

The usual scenario in PHP starts with clinically manifested complications or with an accidental detection of hypercalcemia or of a parathyroid tumour at routine neck ultrasounds. Increased PTH (in addition to high serum calcium) is consistent for the positive diagnosis of PHP while imaging evaluation is required for tumour localization particularly for PTx candidates [71,79,80,87]. Traditional bilateral neck exploration (with a 95% chance of being successful) has been replaced by various pre-operatory imaging techniques from very accessible ones such as ultrasound, computed tomography, and technetium Tc99m parathyroid scintigraphy to more expensive methods as positron emission tomography with radiolabeled 18F-fluorocholine, four-dimensional computed tomography, and sestamibi single-photon emission computed tomography (SPECT) [88,89,90]. Recently, the use of intra-operatory (rapid) PTH testing has become a successful technique in order to predict PHP cure (a 50–60% PTH drop 10 min after parathyroid tumor removal has a high positive predictive value of cure) [91,92]. Of note, the presence of surgical hypoparathyroidism might impact QoL as well [61,65,93]. Self-reported questionnaires as the basis of QoL assessment should be placed at first recognition of PHP, during follow-up under medical therapy or before PTx and after surgery, especially within the first 12 months. A next logical step is an interventional one to correct the components with poor QoL scores, for instance, in deciding (more rapidly) PTx or addressing new lifestyle recommendations for the cognitive and physical components, stress relief techniques, improvement of patients’ support system, particularly in syndromic PHP such as multiple endocrine neoplasia, etc. (Figure 2).

Additionally, other important components might independently damage QoL in patients confirmed with PHP such as chronic renal failure in subjects with a long history of nephrocalcinosis and/or kidney stones that affected the overall function [94,95,96,97,98,99] or the presence of severe (uncorrected) vitamin D deficiency, as we previously mentioned [100]. Also, the presence of osteoporosis, especially severe forms complicated with fragility fractures, might impair the overall mobility and physical functions and correlate with BP; this aspect is more evident in menopausal subjects and older individuals where secondary (PHP-related) reduced bone mineral density and damaged microarchitecture is associated with age- and menopause- associated osteoporosis [101,102,103,104,105].

Generally, various endocrine conditions (from pituitary and adrenal tumors to thyroid malignancies and neuroendocrine neoplasia up to polycystic ovary syndrome or women with infertility seeking to conceive, etc.) had been reported to impact QoL despite not routinely using them in everyday practice [106,107,108,109,110,111].

Our five-year analysis based on sample-study data identified 16 original studies according to our methods: three published in 2023, four—2022, four—2021, one—2020, three—2019, and one in 2018 [12,13,14,17,19,20,24,25,26,27,36,46,64,69,73,74] (Table 1).

A total of 1327 subjects diagnosed with PHP had a QoL evaluation. The studies with the largest number of individuals were of 92 [69], 104 [24], 110 [46], 134 [19], 159 [74], as well as SIPH (which enrolled 95 persons as PTx groups and 96 subjects as non-PTx groups). A few cohorts (*n* = 5) were of small size (between 20 and 40 patients in each of them) [14,17,25,27,36]. Concerning the study design, except for two papers, all the mentioned studies provided longitudinal information, particularly the timeframe from baseline (before PTx) and after surgery. The post-operatory follow-up was of 3–6 months, but mostly between 1 and 3 years (except for SIPH that had a decade of surveillance). 

Apart from one decade-based study [13], Ionova et al. according to their prospective study published in 2023 used a 2-year time frame since surgery [69]. Frey et al. also used a prospective design and repeated QoL analysis after 1 year, respectively, 3 years since parathyroid removal [74], as did Tzikos et al. [25]. Brescia et al. [36], Ejlsmark-Svensson [26], Bannani et al. [20], and Somuncu et al. [14] limited the analysis for the first post-operatory 12 months [14,26,36], 6 months since PTx according to Christensen et al. [17] and Papavramidis et al. [19], and first 3 post-operatory months considering the study of Moh et al. [26]. The exact timeframe of applying the QoL assessment is still an open issue so far. 

The age of the patients varies between medians of 56, 62, 64, and 68 years, with average ages of 40.8 or 50.4 years, respectively, of 62 or 72 years. The most frequent questionnaires were SF-36, PHPQoL, and PAS. Despite not being unanimously similar, an overall reduced score of QoL in patients with PHP versus controls was registered, as well as general improvement following PTx. 

We specifically included, according to mentioned methods, studies on PHP; however, it is well known that specific complications such osteoporosis, osteoporotic (low-trauma or spontaneous) fractures, or kidney stones might impair QoL independently of PTH levels, and these aspects potentially bring a certain level of bias; that is why we did not include them [112,113,114,115,116].

Variations of QoL results might have a multifactorial background from different comorbidities, studied populations, or technical aspects of collecting the data, etc. [12,13,14,17,19,20,24,25,26,27,36,46,64,69,73,74]. Further expansion of this topic and mandatory implementation of these scales in daily practice is yet to be decided. We believe that the most important aspects concern the subjects who are not considered immediate candidates to PTx, whereas QoL assessment might provide useful information in making the decision. 

## 5. Conclusions

According to the data we have so far in PHP, QoL scores depend on numerous parameters like clinical presentation (symptomatic PHP), lab tests (calcium levels), age of the patient, other comorbidities (like MEN syndromes), scales of QoL assessment, timing of post-operatory evaluation, type of surgery, and associated complications. The introduction of a routine evaluation of the QoL scores in patients with PHP, as well as the selection of the most appropriate questionnaire(s), represents an open chapter. It is probable that in the current stage, a selective subgroup of individuals with PHP—e.g., those who are not immediate candidates to surgery—could benefit from this kind of evaluation. QoL scores in PHP may represent a part of the large picture of challenges faced by patients with parathyroid disorders. Meanwhile, modern society targets a deeper understanding of QoL, thereby the field of PHP should not represent an exception.

## Figures and Tables

**Table 1:** Original studies on QoL scores in patients with parathyroid tumors (primary hyperparathyroidism), published between January 2018 and May 2023 (the studies are displayed based on the year of publication, starting with the most recent) [12,13,14,17,19,20,24,25,26,27,36,46,64,69,73,74].

First Author Year of Publication Reference Number	Studied Population Study Design	Quality of Life Assessment	Results
Ionova 2023 [69]	92 patients with PHP (median age of 56 y) prospective study	RAND SF-36 PHPQoL PAS (before PTx + 3, 12, and 24 mo after PTx)	Pre-operatory QoL lower than general population (*p* < 0.001). Post-operatory QoL improvement SF-36 (except for body pain score), and PHPQoL (*p* < 0.001).
Febrero 2023 [73]	65 patients with PHP cross-sectional study	Beck-II and Pittsburg questionnaires SF-36 PHPQoL	Sleep quality was affected in PHP versus controls (*p* < 0.05). Educational level associated with QoL (*p* < 0.05).
Frey 2023 [74]	159 patients with PHP (mean age of 62.6 y) prospective study	SF-36 (before PTx + 1 and 3 y after PTx)	Before surgery: PHP had lower physical and mental components versus controls (*p* < 0.001 for both). After surgery: physical components improved to a similar level with controls, not mental components that improved, but remained lower than controls.
Brescia 2022 [36]	30 patients with MEN1-related PHP (median age of 38 y) prospective study	SF-36 (before PTx + 12 mo after PTx)	Higher PCS, and MCS in asymptomatic vs. symptomatic PHP (*p* = 0.0051, rs. *p* = 0.04) Pre-operatory bone pain correlated with PCS, and MCS (*p* = 0.0162, rs. *p* = 0.0004) Similar PCS, and MCS 6 mo before PTx, rs. 12 mo after PTx QoL is not affected by post-operatory hypoPT, neither by the type of PTx.
Christensen 2022 [17]	40 patients with PHP (median age of 62 y) prospective study	SF-36 (before PTx + 6 mo after PTx)	6 domains of QoL scores improved after PTx: VT (*p* = 0.0001) PF (*p* = 0.038) RP (*p* = 0.043) GH (*p* = 0.004) SF (*p* = 0.004) MH (*p* = 0.0001)
Papavramidis 2022 [19]	134 patients with PHP younger group (≤65 y; N = 95, mean age of 50.4 ± 9.8 y) older group (>65 y, N = 38, mean age of 72.1 ± 4.9 y) prospective study	PAS-Q (before PTx + 6 mo after PTx)	PAS-Q score improvement was after PTx in both groups (but different parameters had the best results among the groups)
Febrero 2023 [64]	65 patients with PHP case control study	SF-36 PHPQoL	QoL, mood, and sleep quality were more affected in patients with PHP vs. controls (*p* < 0.05)
Vadhwana 2021 [12]	74 patients with PHP symptomatic group (N = 56) asymptomatic group (N = 28) prospective study	EQ-5D-3L health status questionnaire (before and after PTx)	Global improvement of VAS score in symptomatic PHP (*p* < 0.001), and asymptomatic PHP (*p* = 0.014) Symptomatic group reduced anxiety/depression levels (*p* < 0.01)
Moh 2021 [26]	50 patients with symptomatic PHP (mean age of 40.8 ± 13.9 y) prospective study	SF-36 (before PTx and 3 mo after PTx)	Overall improvement of QoL when compare the levels before PTx vs. 3 mo post-PTx (*p* < 0.001), especially for BP, RP, VT, and MH (*p* < 0.001) MH scores improvement was dependent of pre-PTx calcium values; PH scores improvement was calcium independent. QoL scores were more affected in patients with severe > mild hypercalcemia
Pretorius 2021 [13]	SIPH (Scandinavian Investigation on Primary Hyperparathyroidism) study PTx group (N = 95) Non-PTx group (N = 96) international, multi-center, 10 y longitudinal, randomized, prospective study	SF-36 CRPS (baseline and after 10 y)	PTx group significantly improved VT score after a decade (*p* = 0.017) Non-PTx group did not change any domain of SF-36 CRPS significantly improved in both groups versus their baseline values, and was similar between the two groups
Somuncu 2021 [14]	41 patients with PHP (median age of 64 y) prospective study	SF-36 PHPQoL (before PTx and 12 mo after PTX)	Both scales showed significant improvement after PTx (*p* < 0.05) 50% for physical domains 76% for MH
Voss 2020 [27]	20 menopausal women with PHP normocalcemic PHP (N = 13) hypercalcemic PHP (N = 7) cross-sectional study	SF-36	GH impairment in first group; MH impairment in second group
Tzikos 2019 [25]	38 patients with asymptomatic PHP PTx group (N = 20) Non-PTx group (N = 18) prospective study	PAS-Q (before PTx + 3 mo after PTx, respective after 3 y)	PTx group: PAS-Q score improved (decreased) after PTx at 3 mo. (*p* = 0.001), respective 3 y (*p* = 0.021) Non-PTx group: PAS-Q score increased after 3 y (*p* = 0.021) Non-PTx group: baseline calcemia correlated with PAS-Q scores (r = 0.524, *p* = 0.018)
Ejlsmark-Svensson 2019 [24]	104 patients with PHP (median age of 64 y) prospective study	PAS-Q PHPQoL (before PTx + 12 mo after PTx)	Ionized calcium and PTH negatively correlated with PHPQoL (*p* < 0.04) PHPQoL was different between subjects with mild vs. moderate-severe hypercalcemia (*p* = 0.01) PAS-Q and PHPQoL were similar between patients with/without osteoporosis/kidney stones/renal dysfunction PAS-Q and PHPQoL were different pre- vs. post-PTX (*p* < 0.02)
Koman 2019 [46]	110 patients with cinacalcet before PTx (mean age of 68 y) retrospective study	QLC-C30 HADSPSMQ MCA	Cincalcet normalized hypercalcemia and correlated with QoL improvement. A subgroup of 11 patients with a baseline score < 26 (mild cognitive impairment) improved the score under cinacalcet (<26), which correlated with post-operatory outcome (PPV = 74–96%)
Bannani 2018 [20]	mild PHP group (N1 = 32) hypercalcemic PHP group (N2 = 82) (mean age of 68 y) longitudinal study	SF-36 (before PTx + 12 mo after PTx)	QoL improvement after PTx for physical domains (*p* = 0.04, respective *p* = 0.016) QoL improvement after PTx for MH component only in hypercalcemic PHP group (*p* = 0.043)

Abbreviations: BP = bodily pain; CRPS = Comprehensive Psychopathological Rating Scale; GH = general health; hypoPT = hypoparathyroidism; HADSPSMQ = Hospital Anxiety and Depression Scale and Positive State of Mind questionnaire; MEN = multiple endocrine neoplasia; MCS = Mental Component Summary; MH = mental health; mo = months; MCA= Montreal Cognitive Assessment; PHP = primary hyperparathyroidism; PTx = parathyroidectomy; PAS-Q = Pasieka’s Parathyroid Assessment of Symptoms Questionnaire; PHPQoL = Primary Hyperparathyroidism Quality of Life questionnaire; PCS = Physical Component Summary; PH = physical health; PF = physical functioning; PPV = positive predictive values; rs. = respective; RP = role-functioning physical; RE = role-functioning emotional; QLC-C30 = European Organization and Treatment of Cancer QLQ-C30 core questionnaire; y = years; vs. = versus; VT = vitality, SF-36 = Short-Form 36 Health Survey Questionnaire; SF = social role functioning; QoL = quality of life.

## Abbreviations

BP	bodily pain
CRPS	Comprehensive Psychopathological Rating Scale
GH	general health
MCS	Mental Component Summary
MH	mental health
QoL	quality of life
PF	physical functioning
PH	physical health
PTH	parathyroid hormone
PCS	Physical Component Summary
PHP	primary hyperparathyroidism
PTx	parathyroidectomy
PAS-Q	Pasieka’s Parathyroid Assessment of Symptoms Questionnaire
PHPQoL	Primary Hyperparathyroidism Quality of Life questionnaire
PROMIS	Patient-Reported Outcomes Measurement Information System
RP	role-functioning physical
RE	role-functioning emotional
SF-36	Short Form 36 Health Survey Questionnaire
SIPH	Scandinavian Investigation on Primary Hyperparathyroidism
SF	social role functioning
SPECT	single-photon emission computed tomography
VT	vitality
VAS	visual analogue scale
